# Changing the degradation footprint of mining on Indigenous Lands

**DOI:** 10.1590/0102-311XEN111223

**Published:** 2023-12-04

**Authors:** Antonio Francisco Perrone Oviedo, Estevão Benfica Senra

**Affiliations:** 1 Instituto Socioambiental, São Paulo, Brasil.; 2 Centro de Desenvolvimento Sustentável, Universidade de Brasília, Brasília, Brasil.; 3 Instituto Socioambiental, Brasília, Brasil.

## Introduction

Illegal gold mining has expanded significantly in the Amazon over the past decade, with an average increase of 7.9% per year. According to data from the 37-year historical series of MapBiomas Collection 7, Brazil’s Legal Amazon covers more than 90% of the mining area in Brazil, and around 12% of the degraded area is Indigenous Lands. In the last 10 years, the area degraded by mining on Indigenous Lands increased by 400%, totaling more than 18,000 hectares, concentrated mainly (90%) in three territories: Kayapo, Munduruku, and Yanomami ^1^.

This expansion was driven by the sharp rise in the price of gold on international markets after the 2008 crisis, the lack of transparency in the production chain and regulatory failures that allow fraud in the declaration of origin of illegally extracted metal, and the weakening of environmental policies and political pressure to revise mining regulations in indigenous territories, led by the governments of formers Presidents Michel Temer (2016-2018) and Jair Bolsonaro (2019-2022).

The Yanomami case has shown that the impacts of mining go far beyond the effects observed in the biophysical environment, such as deforestation and contamination of soil and water resources. The increase in cases of infectious diseases at local level, especially malaria, the intensification of conflicts, and the rise in violence rates also reflect the expansion of the activity ^2^.

Moreover, the harmful effects of illegal mining also go beyond the boundaries of indigenous territories, reaching neighboring municipalities. For example, the negative impacts of mining in the three aforementioned Indigenous Lands affect a population of almost 500,000 individuals (496,797 inhabitants) ^1^.

A study by Oviedo & Araújo ^3^, using data from the Social Progress Index (SPI) for the Amazon ^4^ and the municipalities with Indigenous lands affected by illegal mining, showed that these municipalities have lower social progress indicators compared with municipalities that do not have mining in their territory. Analysis of variance (ANOVA) showed a significant difference between the means at the 5% probability level in the SPI value in 2021 among municipalities with and without mining activities. The 2021 SPI was significantly higher (5.7%) in municipalities without mining. In the municipalities affected by mining, it was also, on average, 20.6% lower than the average index for Brazil ^3^.

The SPI represents the ability of a municipality to meet the basic needs of its citizens, providing the conditions to improve quality of life and environmental sustainability. The results of the aforementioned study show that the municipalities affected by mining have a worse social progress compared with most municipalities in the Amazon, where the average index is already lower compared with other regions of Brazil. The situation is even more critical among the 10 municipalities with the Indigenous Lands most affected by mining (Table 1).

**Table 1:** Social Progress Index (SPI) of municipalities with the Indigenous Lands most affected by illegal mining and its comparison with the average values for municipalities in the Amazon and Brazil.

Municipality (State)	Reduction of the SPI compared with the average for the Amazon (%) (SPI = 54.59)	Reduction of the SPI compared with the average for Brazil (%) (SPI = 63.29)
Jacareacanga (Pará), SPI = 46.83	-14.2	-26.0
Itaituba (Pará), SPI = 53.00	-3.0	-16.3
São Félix do Xingu (Pará), SPI = 53.66	-2.0	-15.2
Bannach (Pará), SPI = 48.89	-10.4	-22.7
Cumaru do Norte (Pará), SPI = 54.14	-1.0	-14.5
Trairão (Pará), SPI = 48.81	-10.6	-22.9
Alto Alegre (Roraima), SPI = 47.87	-12.3	-24.4
Amajari (Roraima), SPI = 47.44	-13.1	-25.0
Caracaraí (Roraima), SPI = 50.15	-8.1	-20.8
Mucajaí (Roraima), SPI = 53.74	-1.6	-15.1

Source: Oviedo & Araújo ^3^.

## How to reverse this socio-environmental degradation footprint?

Some representatives of the Brazilian National Congress and the private sector argue that mining on Indigenous Lands is necessary for the regional development of the Amazon. However, this thesis is not based on solid evidence, quite the opposite. Thus, in line with the study by Oviedo & Araújo ^3^ - which focuses on the areas with illegal mining - other studies that analyze the socioeconomic results of mining activities in the Amazon also show that health and education indicators and gross domestic product (GDP) per capita do not change significantly for the municipalities with this type of exploitation ^5^, despite the huge environmental impacts.

Therefore, stopping illegal mining on Indigenous Lands in the short term is essential. This measure would be favorable for both the protection of Indigenous peoples and the social progress of municipalities with this type of socio-environmental degradation, since, firstly, mining causes unnecessary public expenditure, which could be invested in real improvements in basic services. The expansion of the area degraded by illegal mining, besides increasing the socio-environmental impacts, produces enormous expenses for the health system, public security, social assistance, and environmental inspection ^6^. Secondly, mining is a vector of deforestation and the Amazon rainforest has a growing value and strategic importance for Brazil. The Amazon plays a key role in regulating the climate of the region and the world. A study by Princeton University (United States), for example, showed that the extinction of the Amazon rainforest could lead to a 25% decrease in rainfall in Brazil and an increase in temperature, causing catastrophic losses in the country’s agriculture and energy production ^7^. This region has almost 25% of the above-ground carbon reserves of the world’s forests. If this carbon is released into the atmosphere, global warming would become even more catastrophic. Moreover, Indigenous Lands protect 20.3% of the country’s forests ^8^, contributing greatly to this climate resilience. Finally, the pressure from markets and investment groups to exclude products “contaminated” by mining from foreign trade is enormous. Initiatives such as the Chain-of-Custody Standard of the Responsible Jewellery Council (United Kingdom) ^9^ and the Minerals Due Diligence of the Responsible Minerals Initiative (United States) ^10^ have been developed to assess suppliers’ compliance with legal and socio-environmental mining standards, and have the potential to combat mining.

Article 231 of the Brazilian *Federal Constitution* states that: “*The lands traditionally occupied by Indians are intended for their permanent possession and they shall have the exclusive usufruct of the riches of the soil, the rivers and the lakes existing therein*”. Any legislative proposal to allow mining activities on Indigenous Lands inverts the order of priorities and makes exceptions the order of business.

However, the dynamic of invasions and illegal mining on Indigenous Lands, which has grown in recent years, remains ^11^. According to data from the DETER system (Brazilian National Institute for Space Research - INPE), mining alerts in Indigenous Lands during the first four months of 2023 increased by 93% compared with the same period in 2022. In March and April, for example, the increase in mining alerts was 648.8% and 143%, respectively. This result shows the continuity of invasions and illegal activities inside Indigenous Lands. The analysis of these alerts by the specialists from the Integration Group for the Protection of the Amazon (GIPAM/CENSIPAM) and the Department of Indigenous Environmental and Territorial Rights of the Brazilian National Indian Foundation (FUNAI) is ultimately necessary, allowing for the production of periodic reports to establish consistent inspection operations. If this measure is implemented on a daily basis, the fight can be effective.

Specific actions in the Indigenous Lands most affected by mining should be included in the new Action Plan for Deforestation Prevention and Control in the Legal Amazon (PPCDAm), announced in June 2023 by the Brazilian Ministry of Environment. Among them, we highlight:

(i) Implementing and maintaining ethno-environmental protection bases as territorial control posts and logistical support bases for coordinated operations among the Brazilian Federal Police, the Federal Prosecution Office, the Brazilian Army, FUNAI, the Brazilian Institute of Environment and Renewable Natural Resources (IBAMA), and other relevant bodies to combat illegal mining and other illegal activities.

(ii) Promoting regular operations to destroy clandestine infrastructure and mining support equipment, as well as the immediate expulsion of invaders.

(iii) Blocking the logistics of mining support by river, air, or land, by controlling the rivers that give access to indigenous territories, increasing the strictness of airspace control, destroying clandestine airstrips, and expanding road inspections to seize illegal transportation of fuel, mercury, and illegal timber.

(iv) Promoting strict monitoring of the import, sale, transportation, and use of mercury, in accordance with the terms of the Minamata Convention on Mercury, enacted in Brazil by *Decree n. 9,470/2018* ^12^.

(v) Updating the normative regulations on the supervision of licensed gold mines in the Amazon and the gold trade, to make them stricter and more effective in controlling fraud in the marketing of illegally extracted gold.

(vi) Immediately cancel all mining processes, registered with the Brazilian National Mining Agency (ANM), on Indigenous Lands, including Indigenous territories that are still in the process of demarcation.

(vii) Including equipment and machinery used in mining, deforestation, and selective logging activities (such as backhoes and chainsaws) on the Federal Technical Registry list, with a licensing and control mechanism by IBAMA.

(viii) Developing safe mechanisms so that Indigenous leaders can be constantly contacted and consulted in order to contribute to broad knowledge and action in the monitored areas.

Indigenous communities affected by mining are often fragile and have many difficulties in entering the formal economy in a dignified way. Indigenous peoples hardly participate equally in the hegemonic economy, since they are seen as mere suppliers of raw materials and cheap labor. The concept of the bioeconomy is still a disputed territory and an approach under construction, since it promotes the transition from the current economy, which has waged war on nature, to another paradigm of relationship with nature ^13^.

Thus, the standardization of indigenous peoples’ traditional agricultural systems must be guaranteed as practices for maintaining biodiversity and combating illegal activities. These systems provide benefits associated with: (i) production of sociobiodiversity products and services (products from fields, forest, waters, backyards, community tourism, ecological restoration, etc.); (ii) territorial management practices and biodiversity monitoring; and (iii) promotion and transmission of Indigenous peoples’ knowledge and cultures.

Strengthening this bioeconomy as an antidote to illegal mining requires the formulation of new fiscal, tax, and financial instruments and mechanisms to make sociobiodiversity product chains viable. Existing programs such as the Brazilian National School Feeding Program (PNAE) and the Food Acquisition Program (PAA) are essential to promote traditional agricultural systems, but they need to be adjusted to increase the acquisition of sociobiodiversity products and broaden access for indigenous peoples. For example, measures to adjust the registration of Indigenous individuals and associations, simplify public procurement calls, and allocate budget to agencies for the purchase of products from indigenous peoples and traditional populations are still needed. Moreover, in 2023, the Brazilian National Policy for Territorial and Environmental Management of Indigenous Lands (PNGATI), regulated by *Decree n. 7,747/2012* ^14^ and the Integrated Plan for the Implementation of PNGATI (PII-PNGATI), elaborated by the managing committee of this policy, will be resumed. It is an important instrument for the implementation of actions and goals to be achieved in an integrated manner by government institutions, indigenous civil society organizations, and indigenous organizations.

## Conclusions

The available data shows that environmental degradation caused by illegal mining on Indigenous Lands continues to be high, despite all the warnings and the evidence that this activity does not promote local development. This scenario requires coordination between local and regional policies, strengthening environmental monitoring, promoting chains of sociobiodiversity products, and expanding international mechanisms to increase the traceability of production chains, with certification schemes to curb illegal mining.

Indigenous Lands are essential for the physical and sociocultural reproduction of indigenous peoples and for the exercise of their collective rights. The benefits and services provided by these territories to the climate are undeniable. The right to Indigenous Lands is the central element of constitutional protection. The fundamental rights of indigenous peoples will only be fully guaranteed to the extent that they can combat the socio-environmental impacts on their territories and decide on the essential conditions for their continuity, as they are groups with specific identities and an inseparable part of the Brazilian nation.

Sociobiodiversity product chains are linked to traditional production systems that contribute to the maintenance of landscapes with a very low environmental impact. These practices enable not only greater socio-ecological diversification, but also the continued provision of fundamental ecosystem services for society, such as biodiversity, maintenance of CO_2_ stocks, water, pollination, etc. Recognizing these contributions and services with appropriate public policies is essential to strengthen sociobiodiversity economies and curb the advance of predatory or illegal activities on Indigenous Lands, such as mining, creating mechanisms to promote and value local ways of life in a win-win scenario.
